# Huntingtin Ubiquitination Mechanisms and Novel Possible Therapies to Decrease the Toxic Effects of Mutated Huntingtin

**DOI:** 10.3390/jpm11121309

**Published:** 2021-12-06

**Authors:** Annarita Fiorillo, Veronica Morea, Gianni Colotti, Andrea Ilari

**Affiliations:** 1 Department of Biochemical Sciences “A. Rossi Fanelli”, “Sapienza” University of Rome, P.le A. Moro 5, 00185 Rome, Italy; annarita.fiorillo@uniroma1.it; 2 Institute of Molecular Biology and Pathology of The National Research Council of Italy (CNR), P.le A. Moro 5, 00185 Rome, Italy; veronica.morea@cnr.it (V.M.); gianni.colotti@cnr.it (G.C.)

**Keywords:** ubiquitination, huntingtin, E3 ligases, mutated huntingtin clearance, PROTACs

## Abstract

Huntington Disease (HD) is a dominant, lethal neurodegenerative disorder caused by the abnormal expansion (>35 copies) of a CAG triplet located in exon 1 of the *HTT* gene encoding the huntingtin protein (Htt). Mutated Htt (mHtt) easily aggregates, thereby inducing ER stress that in turn leads to neuronal injury and apoptosis. Therefore, both the inhibition of mHtt aggregate formation and the acceleration of mHtt degradation represent attractive strategies to delay HD progression, and even for HD treatment. Here, we describe the mechanism underlying mHtt degradation by the ubiquitin–proteasome system (UPS), which has been shown to play a more important role than the autophagy–lysosomal pathway. In particular, we focus on E3 ligase proteins involved in the UPS and detail their structure–function relationships. In this framework, we discuss the possible exploitation of PROteolysis TArgeting Chimeras (PROTACs) for HD therapy. PROTACs are heterobifunctional small molecules that comprise two different ligands joined by an appropriate linker; one of the ligands is specific for a selected E3 ubiquitin ligase, the other ligand is able to recruit a target protein of interest, in this case mHtt. As a consequence of PROTAC binding, mHtt and the E3 ubiquitin ligase can be brought to a relative position that allows mHtt to be ubiquitinated and, ultimately, allows a reduction in the amount of mHtt in the cell.

## 1. Introduction

Huntington disease (HD) is an autosomal, dominant, lethal neurodegenerative disorder affecting between 0.42 and 17.2 per 100,000 individuals around the world [1,2,3] HD results in a wide range of symptoms, including involuntary movements, clumsiness, lack of concentration, memory lapses, mood swings, and depression. Although brain pathology is considered a hallmark of HD, new studies suggest that peripheral tissue pathology is an important factor in disease manifestation and progression. In particular, HD mouse models have recently been shown to display skeletal muscle malfunction and HD-related cardiomyopathy [4,5,6].

Primary symptoms generally occur in adults (typical age range: 40–45 years) and gradually progress, leading to the deterioration of patients’ health, and finally death, after 10–20 years. Although several clinical trials are ongoing, there is no cure for HD at present. HD is caused by a mutation in the *HTT* gene, which encodes for the huntingtin (Htt) protein. The *HTT* gene contains a repeat of 6–35 CAG triplets in exon 1, which is translated into a polyglutamine (polyQ) stretch in the Htt N-terminal region. The HD patients’ gene contains ≥36 CAG triplets, encoding for a mutated Htt (mHtt) with an expanded polyQ region [7]. mHtt is highly susceptible to aggregation with other mHtt molecules or different proteins, leading to the formation of clusters, fibrils and inclusions, some of which comprise 100,000s of mHtt molecules and are large enough to be visualized by light microscopy [8,9].

CAG stretches that are longer than 60 repeats in the Htt gene are associated with Juvenile-onset Huntington Disease (JoHD). This is a rare HD variant that accounts for about 4–10% of all cases, typically defined based on the appearance of symptoms at age 20 years or younger [10]. JoHD patients experience different motor and non-motor symptoms at disease onset and throughout the disease course, with a faster disease progression rate and reduced life span with respect to adult HD patients [11].

Since both soluble and aggregated mHtt are well known to induce ER (Endoplasmic Reticulum) stress, leading to neuronal injury and apoptosis [12], both the inhibition of mHtt aggregate formation and the acceleration of mHtt degradation could be exploited for HD symptom delay, or even treatment [13]. mHtt can be degraded by two main pathways: the ubiquitin–proteasome system (UPS) and the autophagy–lysosomal pathway. In this paper, we focus on the UPS, since it has been shown to play a more important role in removing mHtt than the autophagy–lysosomal pathway [14]. In this framework, it is important to underline that cells contain a variety of molecular chaperones and other proteins, such as heat shock family proteins HSP40, HSP70, HSP90, and HSP105, which are able to identify and combine with misfolded mHtt to inhibit aggregate formation to different degrees, leading to cell survival [15,16,17,18,19].

Here, we describe the mechanism of mHtt ubiquitination, with particular emphasis on the structure–function relationships in E3 ligases involved in the process. Finally, we discuss how knowledge of these relationships can be exploited to develop PROteolysis TArgeting Chimeras (PROTACs), which may be used to develop innovative drugs able to increase mHtt clearance in HD patients.

## 2. Ubiquitin Proteasome System (UPS)

The UPS comprises ubiquitin (Ub), proteasome, and three classes of enzymes, and plays key roles in various essential biological processes, such as cell cycle progression, signal transduction, maintenance of genome integrity, and tumorigenesis [20].

**Figure 1:**
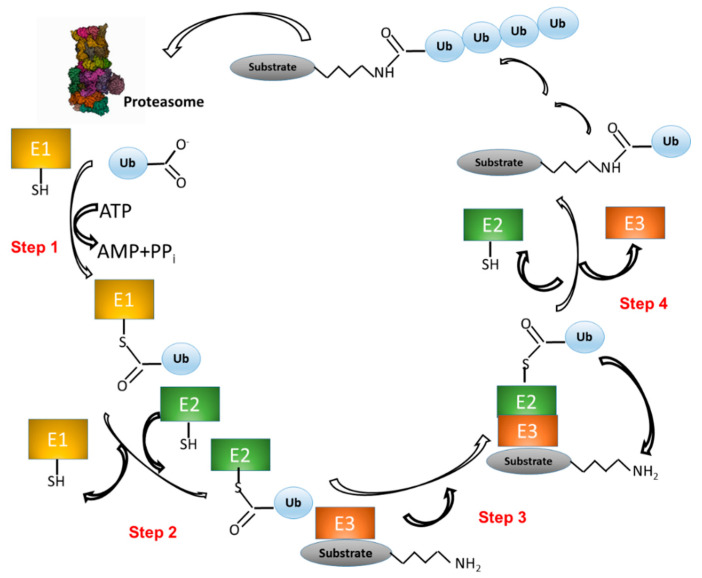
The ubiquitination pathway. (1) Ubiquitin (Ub) is linked to a cysteine residue of a Ub-activating enzyme (E1) via a reaction that uses the energy derived from the hydrolysis of an ATP molecule. (2) Ub is transferred from E1 to the cysteine of a Ub-conjugating enzyme (E2). (3) A complex is formed between E2–Ub and a Ub ligase (E3), which is able to bind a protein substrate. (4) Ub is transferred from E2 to a lysine residue of the protein substrate. Other Ub molecules can be added to the substrate–Ub complex with the same mechanism (steps 1–4). Finally, the polyubiquitinated substrate is recognized and degraded by the proteasome, whereas Ub is released and ready to start a new cycle.

UPS activity consists of two main processes: (1) covalent attachment of multiple Ub molecules to the target protein; and (2) degradation of the resulting covalent target protein–Ub complex by the 26S proteasome complex (Figure 1). The first process, in turn, encompasses several steps and requires at least three classes of enzymes: (i) Ub-activating enzymes (E1); (ii) Ub-conjugating enzymes (E2); and (iii) Ub ligases (E3). In the first step, E1 enzymes use ATP to activate the Ub carboxy-terminal region (C-ter). The resulting Ub–AMP drives the formation of a thioester bond between Ub and a cysteine residue of E1. In the second step, Ub is transferred to a cysteine residue of an E2 enzyme. In the third step, the E3 ligase binds both the Ub–E2 adduct and the target protein. In step four, an isopeptide bond between Ub and the protein target is formed (Figure 1) [21,22]. The process that consists in the linkage of a protein to a single Ub monomer is known as monoubiquitination.

**Figure 2:**
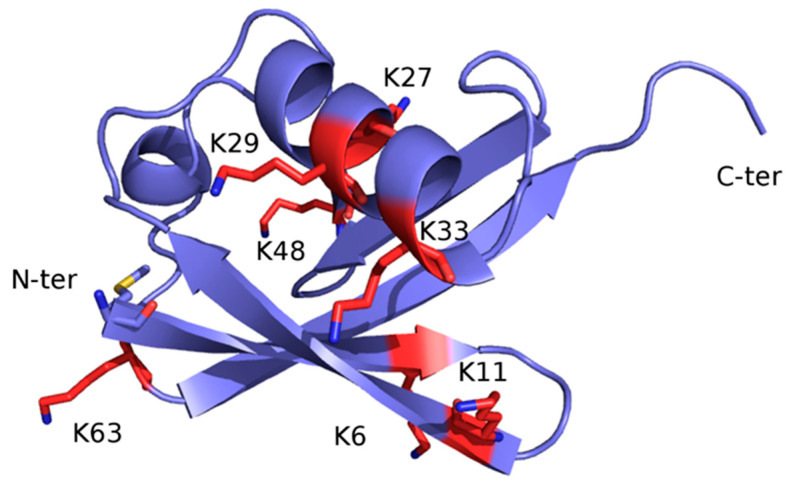
X-ray structure of Ub (PDB code: 1UBQ). The structure is represented as ribbon and colored light blue. The seven lysine residues are represented as sticks and colored red, except for the side chain amino group, which is blue. The N-terminal methionine residue (M1) is shown as sticks and colored by atom type: blue, N (of the main-chain free amino group); red, O; yellow, S; light blue, C.

Once monoubiquitination has occurred, the Ub moiety can be ubiquitinated again at one of the seven lysine sidechains (K6, K11, K27, K29, K33, K48, and K63) or at the free main-chain amine group of the N-terminal methionine residue (M1) (Figure 2), thus extending the modification into a polyubiquitin chain. The ability of Ub to link other Ub molecules at eight different sites is the molecular basis of a dynamic and complex system regulating cellular metabolism. In turn, the different chain types resulting from polyubiquitination processes dictate the specific signaling function associated with the modification [23,24,25].

Polyubiquitin linkages are classified as: (i) homotypic, if Ub molecules are bound to a single lysine position of the substrate; (ii) heterotypic, in case Ub molecules are bound at different lysine positions of the substrate; (iii) branched, when ubiquitination takes place at two or more sites on a single Ub molecule [26]. Ub exerts several specific functions, which are contributed not only by monoubiquitin, but also by numerous different combinations of polyubiquitin linkages.

Monoubiquitination contributes to several processes, including DNA repair, control of transcription, metabolism, and apoptosis [27].

The role of homotypic chains depends on the position of the lysine residue involved in the linkage. K48 Ub, wherein a Ub chain is covalently bonded to the ε-amino group of the lysine at position 48 of the preceding Ub molecule, is the most abundant linkage type in homotypic polyubiquitin chains and represents the canonical signal for proteasomal degradation [28]. K11 Ub is particularly abundant as well [26] and has proteasome-independent functions, including intracellular signaling [29,30], in addition to proteasomal-dependent degradation [31]. K63 Ub regulates several processes, including the multiple translation process independent of the proteasome. This encompasses translation quality control, in particular during oxidative stress, and is also known to induce autophagy [27,32,33].

The heterotypic, branched K48 and K11 are associated with proteasomal degradation of several cell-cycle regulators, including cyclin B1 and securin, thereby promoting mitotic exit.

Currently, the functions of other Ub linkages are beginning to emerge.

### 2.1. E1-Activating and E2-Conjugating Enzymes

There are only two human E1s (Ub-activating enzymes; see Figure 1), i.e., UBA1 and UBA6. UBA1 is the most studied E1 and one of the most abundant proteins in humans. It is expressed at a level of about one order of magnitude higher than UBA6 [27] and is responsible for most protein ubiquitination in humans. The first crystal structure of a UBA1–Ub complex has recently been solved [34]. Using structural analysis, molecular modelling, and biochemical analysis, the authors demonstrated that UBA1 shares a conserved overall structure and catalytic mechanism with previously characterized yeast orthologs while displaying small differences in the active site. Moreover, structural analysis revealed four potential regions that might be targeted in PROTACs-based approaches (see below) [34].

Humans possess ~40 E2s (Ub-conjugating enzymes; see Figure 1). The structures of over 32 human E2 proteins have been solved. They share a common core catalytic domain, called the UBC domain, which comprises 150 residues and adopts a conserved α/β-fold. This typically contains four α-helices and a four-stranded β-sheet, as well as important loop regions that are part of the E2 active site or E3-binding site [35].

### 2.2. E3 Ligases

E3 (Ub ligases; see Figure 1) components in the UPS are responsible for specific recognition of a large number of target proteins. To date, 377 E3 Ub ligases have been identified in the human genome (https://hpcwebapps.cit.nih.gov/ESBL/Database/E3-ligases/, accessed date 1 February 2021), with a total estimated number of over 600 E3 ligase genes, representing ~3% of the human genome [36]. These have been assigned to three major classes on the basis of the structural motifs in their catalytic domain: (i) Homologous to E6-AP Carboxyl Terminus (HECT)-type; (ii) Really Interesting New Gene (RING)-type; and (iii) U-box-type [37].

**Figure 3:**
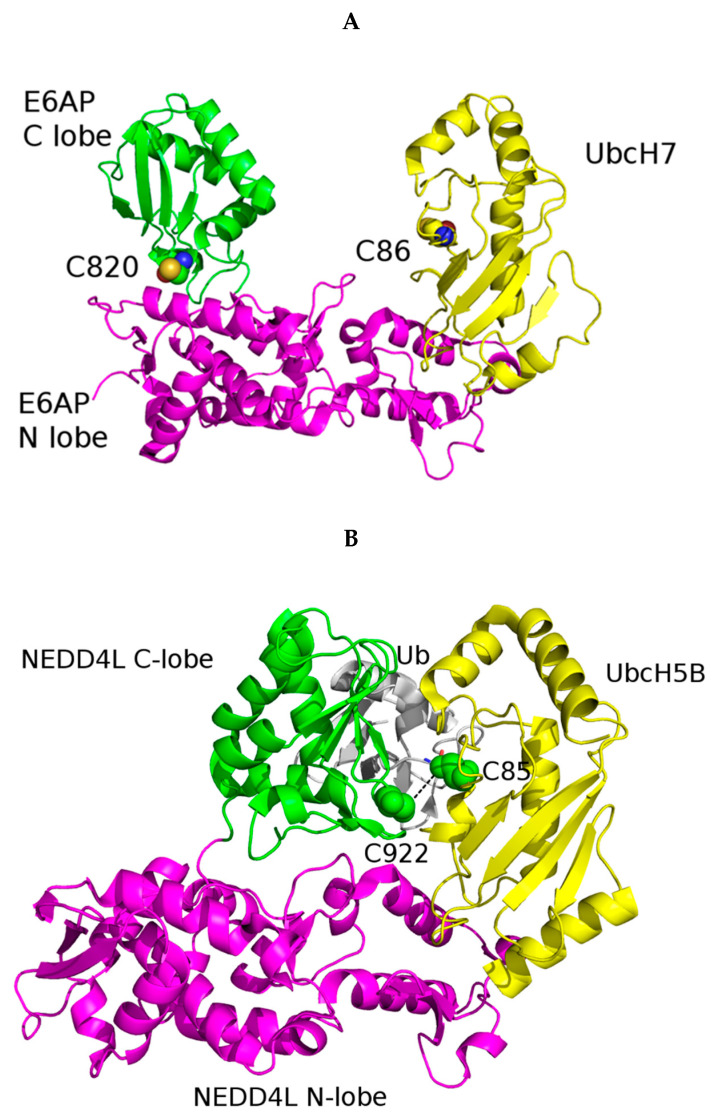
(**A**). HECT domain of UBE3A/E6AP in complex with the E2 UbcH7 (PDB code: 1C4Z). UbcH7 is colored yellow and the C and N lobes of the HECT domain are colored green and magenta, respectively. The catalytic cysteine residues of UBE3A and UbcH7 are represented as spheres and colored by atom type (N, blue; O, red; S, yellow; C, green and yellow, like the respective ribbon color). (**B**). HECT domain of NEDD4L in complex with the E2 UbcH5B (PDB code: 3JW0). Color coding: UbcH5B, yellow; C and N lobes of the NEDD4L HECT domain, green and magenta, respectively; ubiquitin (Ub), grey. The catalytic cysteine residues of NEDD4L and UbcH5B are shown as spheres and colored green.

In mammals, there are ~30 HECT E3 ligases. These proteins are involved in protein trafficking, immune response, and several signaling pathways that regulate cellular growth and proliferation [38]. HECT-type E3 ligases are named after the conserved C-terminal HECT domain. The N-terminal domain is not conserved because it serves to bind different target proteins. The conserved HECT domain comprises ~350 amino acids and two lobes. The N-terminal N-lobe is deputed to the interaction with E2, and the C-terminal C-lobe contains the catalytic cysteine able to form a thioester bond with Ub [39]. In the structure of the complex between the E6-associated protein (E6AP, also known as UBE3A), the first identified member of the HECT family, and the E2 UBCH7 (also known as UBE2L3), the C-lobe of UBE3A acquires an open conformation wherein the E2 and E3 catalytic cysteines are 41 Å apart (Figure 3A) [40]. In contrast, in the crystal structure of the E3 Ub protein ligase NEDD4-like (NEDD4L) HECT domain in complex with the Ub-conjugating enzyme E2 D2 (UBE2D2, also known as UbcH5B), NEDD4L acquires a compact structure that wraps around the UBE2D2–Ub complex, wherein the E2 and E3 catalytic cysteines are close to each other (~8 Å between the reactive S atoms) (Figure 3B) [41].

The RING-type E3 ligases contain a Zn^2+^-coordinating domain that binds the E2 ligase, and another domain able to bind the target protein, thereby facilitating the E2-dependent ubiquitylation of the target protein. The structure of the RING domain comprised in the c-CbI portion (residues 47 to 447) in complex with UBCH7 E2 illustrates several notable features of the RING domain [42]. In particular, the two zinc ions and the coordinating residues form a ‘cross-brace’ structure that brings E2 and the substrate together. Members of the RING finger E3 ligase family can function as monomers, dimers, or multi-subunit complexes. These proteins can form either homodimers or heterodimers. Homodimers include: cellular inhibitor of apoptosis (cIAP), officially known as BIRC2; RING finger protein 4 (RNF4); seven in absentia homologue 1 (SIAH); and TNF receptor-associated factor 2 (TRAF2). Examples of heterodimers are: murine double minute 2 (MDM2), also known as HDM2 in humans and murine double minute X (MDMX, officially known as MDM4, and also known as HDMX or HDM4 in humans); breast cancer 1 (BRCA1) and BRCA1-associated RING domain 1 (BARD1); RING1b (officially known as RNF2) and B cell-specific Moloney murine leukemia virus integration site 1 (BMI-1) and polycomb RING finger oncogene (BMI1). Very often, in heterodimers, RING domain-containing proteins (such as MDMX, BARD1, and BMI1) do not act as ligases, but stabilize the active E2-binding RING domain.

The cullin RING ligase (CRL) superfamily comprises multi-subunit RING domain-containing proteins. One of the members of this superfamily is the SCF complex, consisting of S-phase kinase associated protein 1 (SKP1), cullin and F-box protein, and the anaphase promoting complex/cyclosome (APC/C) [39].

The U-box-type E3 ligases contain a domain of 70 amino acids that is conserved in proteins from yeast to humans and has been identified in yeast for the first time (Ufd2). Ufd2, the prototype U-box protein, is a Ub chain assembly factor (E4) that cooperates with one E1, one E2, and one E3 enzyme to catalyze the conjugation of a Ub chain to substrates. Mammalian U-box proteins, in conjunction with E1 and E2, are also able to polyubiquitylate proteins, facilitating the transfer of Ub from E2 to the target in the absence of HECT-type or RING-type E3 ligases. For this reason, they can be considered to be a third family of E3s [43].

### 2.3. Deubiquitinating Enzymes

Deubiquitinating enzymes (DUBs) remove Ub molecules thanks to their Ub C-terminal hydrolytic activity. The human genome encodes ∼100 DUBs, which are divided into six families: Ub-specific proteases (USPs); Ub C-terminal hydrolases (UCHs); ovarian tumor proteases (OTUs); Machado–Joseph domain-containing proteases (MJDs); JAB1/MPN/MOV34 family (JAMMs); and motif interacting with Ub-containing novel DUB (MINDY) family. All enzymes belonging to these families, with the exception of the JAMM zinc metalloproteases, are cysteine proteases [23].

### 2.4. Degradation though the Proteasome 26 S

The 26S proteasome is the major proteolytic machine in eukaryotic cells. It is responsible for protein degradation in both cytosol and nucleus. It degrades target proteins that have been covalently modified by ubiquitination. Ub molecules attached to lysine side chains are removed at the proteasome prior to substrate degradation [44]. The proteasome exploits the energy deriving from ATP hydrolysis to first disrupt the quaternary and tertiary structures of the protein substrate, and then transfer the unfolded protein into an internal degradation chamber where it will undergo proteolytic cleavage. This ability allows the proteasome to function as a modulator of the eukaryotic proteome and degrade not only damaged or misfolded polypeptides but also many regulatory proteins. As a consequence, the role of 26S proteasome is not limited to protein and amino acid homeostasis maintenance, but extends to the regulation of cellular processes including DNA replication, transcription, signal transduction, response to stress, cell cycle, etc. [44]

The high selectivity and control of protein degradation are guaranteed by the specific Ub labelling of protein substrates and by the architecture of the 26S proteasome holoenzyme. The proteasome is formed by a barrel-shaped 20S core particle, which contains the proteolytic active sites, and a 19S regulatory particle, which caps one or both ends of the core particle. Ubiquitinated substrates are recognized by Ub receptors RPN1, RPN10, and RPN13. To access the proteolytic sites, protein substrates have to penetrate narrow axial pores, which cannot be reached by folded or even largely unfolded polypeptides. The regulatory particle controls the opening of pores and favors substrate translocation into the degradation chamber. Substrate globular domains are mechanically unfolded by a ring-like heterohexameric adenosine triphosphatase (ATPase) motor. This consists of six distinct subunits (RPT1–RPT6) from the ‘ATPases associated with diverse cellular activities’ (AAA+) family and regulates the engagement, the RPN11-catalysed deubiquitination, and the degradation of substrates in an ATP-dependent manner through a mechanism that is still partially unknown [45,46].

## 3. The Level of Huntingtin Is Controlled by Ubiquitination

### 3.1. Htt Structure and Function

In HD patients, the polyQ region located after the first seventeen N-terminal Htt residues is expanded beyond a threshold of 36 glutamine residues (mHtt) [46].

A distinctive feature of HD is the progressive death of striatal projection neurons (SPNs), which are GABAergic output neurons representing >90% of striatal cells. SPNs are divided into two groups, depending on whether they belong to the direct (DP) or indirect (IP) pathway (DP-SPNs and IP-SPNs, respectively). Both SPN sub-types receive extensive glutamatergic inputs from cortex and thalamus, and dopaminergic inputs from the ventral tegmental area and substantia nigra pars compacta [46].

Within neurons, mHtt molecules form toxic aggregates with a rate proportional to the length of the polyQ expansion. mHtt co-aggregates with a number of different proteins, which decreases their concentration in the cell. Several studies demonstrate that polyQ expansion in mHtt results in a toxic gain-of-function phenotype. Other studies, which include gene knockouts and knockdowns, demonstrate that polyQ expansion in mHtt can also have loss-of-function effects. The role of wild-type Htt should therefore be taken into account in the development of a potential therapy, as well as that of mHtt [47].

The structure of Htt in complex with HAP40 has been recently solved by cryo-EM at 4 Å resolution [17]. Conversely, unbound Htt is very difficult to study either by X-ray crystallography or cryo-EM because it has a large size (it contains 3144 residues and weights 348 kDa), is very flexible, and tends to form aggregates. However, about 28% of Htt residues are not visible in the structure, including the first 91 residues, which comprise the 17 glutamine residue-containing polyQ expansion. In agreement with computational predictions, all secondary structure elements belonging to either Htt or HAP40 resolved in the model are α-helices, most of which (72%) are arranged in HEAT or other tandem repeats.

**Figure 4:**
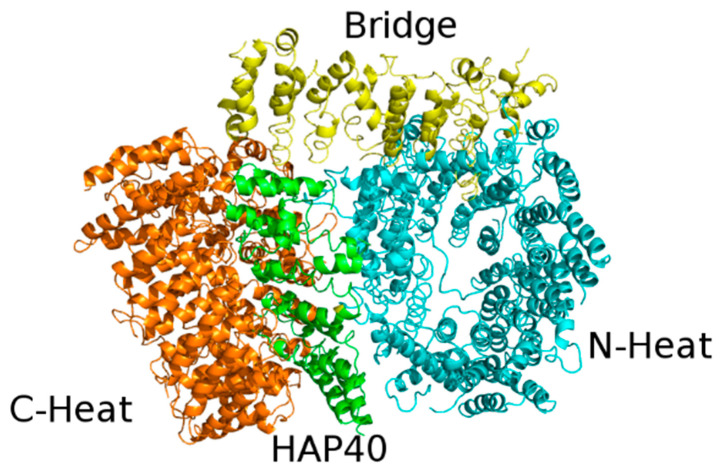
X-ray structure of the complex between HAP40 and Htt (PDB code: 6EZ8). HAP40 is colored green and the C-Heat, Bridge, and N-Heat domains of Htt are colored orange, yellow, and cyan, respectively.

The cryo-EM structure revealed that Htt is formed by three domains: N-terminal domain and C-terminal domain, both of which contain multiple HEAT repeats, joined to a bridge domain (Figure 4).

The N-terminal domain (N-HEAT; residues 91–1684) forms a typical α-solenoid, comprising 21 HEAT repeats arranged as a one-and-a-half turn, right-handed superhelix. The N-HEAT was predicted to contain two membrane binding regions: the 1–17 N-terminal tail (not visible in the structure), which was predicted to form an amphipathic helix [48], and a larger region (comprising a.a. 168–366), containing a functionally important palmitoylation site at Cys208 [49]. This region (N-HEAT repeats 2–4) is placed on the N-HEAT convex surface and is positively charged.

The C-HEAT (a.a. 2092–3098) comprises 12 HEAT repeats that form an elliptical ring of ~80 × 30 Å.

The N-HEAT and C-HEAT are joined by the bridge domain. This contains six tandem α-helical repeats, of which repeats three, four and six are Armadillo-like. The repeat region is flanked by five non-repeat helices and a flexible C-terminus (a.a. 2062–2092), which is unresolved.

The lack of interactions between the N-HEAT and C-HEAT domains explains the flexibility of the Htt structure in the absence of interactors. HAP40 binds to a large cleft between these two domains. Within the complex, Htt and HAP40 share large interfaces that comprise mostly hydrophobic interactions. Interestingly, bioinformatic analyses has provided evidence of the evolutionary conservation of these interfaces in all Htt-encoding animal species [50].

Recently, the structure of wild-type Htt in complex with HAP40 has been compared with other mHtt–HAP40 complexes differing in the length of the polyQ repeat, namely, 46QHtt–HAP40 (46 being a typical polyQ length in HD patients) and 128QHtt–HAP40 (128 being an extremely high polyQ length). Quite surprisingly, both crosslinking mass spectrometry and cryo-EM experiments revealed no major structural differences among the different complexes, indicating that the polyQ insertion does not alter the Htt fold [51].

Htt function has not yet been fully elucidated, but Htt has been proposed to be involved in cellular processes such as mitotic spindle orientation, autophagy, and vesicle transport [52,53,54,55]. Htt has been shown to be essential in both embryonal development and adult life. In mice, the Htt knockdown mutant dies at about day 8.5 of gestation [56]. Additionally, Htt deletion in the mouse central nervous system leads to a phenotype similar to that of HD, i.e., cellular stress, neuroinflammation, aberrant synaptic connectivity, and neuronal death [57,58,59].

Htt is crucial for energetic metabolism not only in the brain [60] but also in peripheral tissues. In Htt-null cardiomyocytes, both intracellular ATP and total purine concentration in the cellular medium were reduced. This indicates that, in the heart, Htt plays an important role in both cellular energy balance and nucleotide metabolism [61].

Recent studies carried out using mouse embryonic stem cell demonstrated that Htt is necessary for mitochondrial structure and function from the earliest stages of embryogenesis, providing a molecular explanation for the early embryonic lethality of Htt knockdown [62].

SPN death in HD has mostly been ascribed to toxic ‘gain-of-function’ by mHtt. However, evidence of Htt ‘loss-of-function’ contribution is also available. Indeed, wild-type Htt has been shown to be neuroprotective and, therefore, able to shield neurons against mHtt toxicity [63]. Additionally, Htt deletion in IP-SPN and DP-SPN leads to a phenotype that resembles the key features of HD, supporting the hypothesis that Htt loss-of-function contributes to SPN pathology in HD [64]. Lowering of wild-type Htt expression has also been shown to affect both health and function of primary monocyte-derived macrophages from healthy human subjects, likely by different mechanisms with respect to those associated with mHtt [65].

### 3.2. Htt Ubiquitination and SUMOylation

Htt is a protein with high ubiquitination potential. It contains 124 lysine residues, many of which are placed on the protein surface, as shown by cryo-EM structural analysis, and may in principle be ubiquitinated or linked to other Ub-like (Ubl) proteins, such as SUMO (Small Ub-like Modifier).

Ubiquitination and/or SUMOylation has been demonstrated for the 30 Htt lysine residues reported in Table 1. Ubiquitination and SUMOylation processes have been shown to compete for lysine residues K6, K9, and K15, all of which are placed in the N-terminal tail that is not visible in the solved cryo-EM structure [66], and they may compete for other lysine residues as well.

Ubiquitination has been linked to reduced mHtt toxicity, most likely due to increased mHtt clearance by the proteasome [74]. Conversely, SUMOylation has been shown to stabilize mHtt, reduce mHtt aggregation, enhance transcriptional dysregulation by mHtt, and increase mHtt toxicity in a Drosophila model [66]. SUMOylation contribution to mHtt toxicity may be mediated by mHtt targeting of the nucleus and the hampering of ubiquitination and subsequent degradation. While Htt ubiquitination is mediated by different E3 ligases, mHtt SUMOylation is mediated by only one protein, i.e., RHES (Ras homolog enriched in striatum) [75]. RHES has higher affinity for mHtt than wild-type Htt, and its selective expression in the striatum strongly suggests that this protein contributes to the HD pathology [76]. For these reasons, RHES has been considered to be an attractive target for HD therapy [77].

As described above, the presence of seven lysine residues allows Ub to transmit different signals on target proteins. Therefore, Htt polyubiquitination can take place with different mechanisms, which have not yet been completely understood. Two among the 40 E2 enzymes present in the human genome have been demonstrated to interact with Htt: UBE2W, which is known to target the N-termini of disordered proteins such as Tau, RBPB8, and ATXN3, as well as mHtt [78]; and UBE2K, also known as Htt-interacting protein (Hip2) or E2-25k, which is a Ub-conjugating enzyme that directly interacts with Htt and may mediate its ubiquitination [79].

A variety of E3 Ub ligases have been shown to act on polyQ-containing proteins such as mHtt, thereby determining a reduction in mHtt cellular levels, aggregation, and cellular toxicity. Some of these E3 Ub ligases are able to directly polyubiquitinate Htt via K48 of Htt-linked ubiquitin, allowing its clearance by the UPS. These include UBE3A/E6AP [80,81], CHIP [82], HRD1 [83], Parkin [84], and the SCF complex [85]. Other E3 ligases, such as HACE1, counteract the negative effect of mHtt on redox metabolism [86]. Other E3 ligases, such as WWP1 [87] and TRAF6 [88], which are upregulated in the postmortem brains of people with HD, mediate atypical Htt ubiquitination (at K6, K27, and K29), thereby inhibiting mHtt degradation and favoring the formation of ubiquitinated aggregates.

HD has been associated with global changes in the UPS, such as the accumulation of K48- K63- and K11-linked polyUb chains, and the inhibition of proteasome activity by mHtt has been proposed to contribute to neurotoxicity, although the molecular basis of this process is not clear. The autophagic pathway has been involved in Htt aggregate clearance, and autophagy upregulation has been shown to reduce polyQ expansion toxicity in HD mouse models [89].

Only a few DUBs have been reported to deubiquitinate Htt. ATXN3, which contains a polyQ region itself, and USP19 are the only DUBs known to target Htt besides proteasome-associated DUBs [90,91].

## 4. E2-Conjugating Enzymes Interacting with mHtt and Htt

### 4.1. UBE2K/E2-25k Triggers polyQ-Induced Cell Death

UBE2K, also known as the Ub-conjugating enzyme E2-25K, was reported to be expressed in all areas of the brain, with higher levels in the striatum and frontal cortex, which are among the most affected areas in HD patients [74]. UBE2K is known to interact directly with mHtt and might therefore modulate mHtt aggregation and toxicity. UBE2K is involved in the formation of expanded polyQ-containing protein aggregates and in polyQ-induced cell death [79]. The influence of UBE2K on aggregate formation and cell viability was tested using lentiviral vectors containing: the RNA codification for the full-length enzyme; a UBE2K variant lacking the catalytic C-terminal domain (UBE2K deletion mutant); and the complete antisense mRNA. The UBE2K deletion mutant was not able to catalyze Htt polyubiquitination but was still able to interact with the Uba1–Ub complex, decreasing its availability for the ubiquitination process. UBE2K expression in cell lines was knocked down by the antisense UBE2K mRNA [92]. Lentiviral transduction of SH-SY5Y neuroblastoma cells with polyQ vectors in combination with one of the UBE2K vectors showed that both the truncated mutant and the full antisense sequence reduce aggregate formation and polyQ-induced cell death. This means that UBE2K-mediated ubiquitination not only influences the formation of expanded polyQ protein aggregates but, most importantly, triggers polyQ-induced cell death.

### 4.2. UBE2W Ubiquitinates mHtt at the N-Terminus

UBE2W (Ub-conjugating enzyme 2W) is the only E2 enzyme known to be involved in the ubiquitination of substrates at their N-termini, likely preferring substrates with disordered N-termini such as mHtt. This conjugating enzyme can function with various Ub ligases, including the C-terminus of Hsc-70-interacting protein (CHIP) [78].

In cells, mHtt exists in at least three distinct states: soluble monomers; soluble oligomers; and insoluble aggregates. Soluble oligomers have been demonstrated to be more toxic than monomers and insoluble aggregates [93].

In studies conducted on immortalized cells, primary neurons, and a HD knock-in mouse model, UBE2W deficiency resulted in a reduced formation of mHtt oligomers and an increased formation of soluble mHtt monomers and neuron survival. The reason for this behavior is still unknown, but the most accredited hypothesis is that mHtt ubiquitination increases protein aggregation and/or impedes other post-translational modifications such as SUMOylation, which takes place on the same mHtt lysine residues as ubiquitination [78].

## 5. E3 Ligases Decreasing mHtt Levels in HD

### 5.1. UBE3A Structure and Its Role in mHtt Ubiquitination

UBE3A, also known as E6AP, is a HECT-type E3 ligase. The crystal structure of UBE3A catalytic HECT domain was solved both in the apo form (PDB accession code: 1D5F; resolution: 2.8 Å) and in complex with the UBCH7 conjugating enzyme fragment comprising residues 495 to 852 (PDB accession code: 1C4Z; resolution: 2.6 Å) (Figure 3A). The HECT domain is a 40-kD carboxy-terminal catalytic domain that performs four main functions: (i) specific E2 binding; (ii) formation of a thioester intermediate with a Ub molecule transferred from the E2 ligase; (iii) Ub transfer to the ε-amino groups of lysine side chains on the protein substrate and catalysis of isopeptide bond formation; (iv) transfer of additional Ub molecules to the growing end of the multi-Ub chain [40,94].

UBE3A plays a critical role in Htt ubiquitination. This role is modulated by UBE3A levels of expression, which decreases with age. UBE3A associates with mHtt in the striatum of a HD knock-in mouse model. Co-immunoprecipitation assays in HEK293 cells transfected with several N-terminal Htt fragments showed that UBE3A binds higher amounts of mHtt than wild-type Htt, and, in particular, it binds the shortest mHtt fragment N67-150Q (comprising Htt N-terminal 67 residues and containing a 150 residue polyQ repeat). UBE3A expression levels decrease with aging and may be further downregulated by the presence of mHtt. As a result, the decrease in UBE3A expression levels occurring in the aged HD knock-in mouse model causes an impairment of the mHtt degradation process that takes place via K48 ubiquitination. Conversely, K63-ubiquitinated mHtt and mHtt fragment levels increase and lead to the formation of aggregates that give rise to the K63 immunoreactive signal in the striatum. On the other hand, when HEK293 cells transfected with N-terminal mHtt fragments (N67-150Q, N212-120Q, and N508-120Q) are transfected with UBE3A as well, the level of these short mHtt fragments decreases, indicating that they are degraded via UPS [95].

All together, these data indicate that UBE3A increases the levels of mHtt and mHtt fragment ubiquitinated via K48 and degraded via UPS; with aging, UBE3A levels in the cell decrease and, as a consequence, the aggregates of mHtt increase.

### 5.2. CHIP Inhibits PolyQ Protein Aggregation

The C-terminus of Hsc70-interacting protein (CHIP) is a U-box E3 ligase whose N-terminal tetratricopeptide repeat (TPR) regions bind HSP70, HSC70, and HSP90 chaperones and potentially target any chaperone-bound protein for degradation [82]. CHIP is able to ubiquitinate polyQ expanded proteins such as mHtt and ataxin-3 [96]. CHIP overexpression inhibits polyQ protein aggregation and cell death, an effect that is particularly evident when CHIP is expressed along with the Hsc70 chaperone.

### 5.3. Hrd1 Protects Cells against Cell Death Induced by mHTT N-Terminal Fragment

Hrd1 is an endoplasmic reticulum (ER), membrane-spanning, RING finger, E3 ubiquitin ligase whose catalytic domain faces the cytosol and can ubiquitinate misfolded proteins therein. Hrd1 levels were found to be increased in cells overexpressing the 588-residue N-terminal fragment of Htt containing an expanded (138 residues) polyQ region (HttN). HttN was stabilized by Hrd1 expression silencing by RNA interference, whereas Hrd1 forced expression enhanced the degradation of HttN. Hrd1 was demonstrated to interact with and ubiquitinate HttN, recruit HttN to the ER, and co-localize with juxtanuclear aggregates of HttN in cells. Hrd1 interacts with HttN fragments comprising polyQ region of different length, with a preference for longer polyQ regions, and targets pathogenic HttN for degradation and protects cells against HttN-induced cell death [83].

### 5.4. Parkin Suppression Aggravate Motor and Behavioural Deficits in HD Mice

Parkin (PK) is a RING-between-RING E3 ligase that possesses both a canonical RING domain and a catalytic cysteine that is characteristic of HECT E3 ligases. For this reason, it is also termed as a ‘RING/HECT hybrid’ [97].

PK promotes proteasomal degradation of abnormal proteins. The effect of partial PK suppression on HD phenotype was investigated by studying the behavior and brain histology and biochemistry of wild-type, R6/1 (mice with mHtt containing about 115 CAG repeats), R6/1/PK+/−, and PK+/− mice. PK partial suppression did not cause additional neural loss either in striatal regions or other parts of the brain, indicating that the lower expression of PK is partially compensated by other protection mechanisms. However, R6/1/PK+/− mice have a higher level of apoptotic cells in hippocampus and striatum with respect to the R6/1 mouse, as well as a higher Bax/Bcl2 ratio. This increment in apoptotic cells results in an exacerbation of motor and behavioral deficits in R6/1 mouse models [84].

### 5.5. SCF Complex

The Skp, Cullin, F-box-containing (SCF) complex is an E3 Ub ligase [98]. The X-ray structures of human SCF show that it contains three core subunits, i.e., RING protein Rbx1, Cul1, and Skp1-F boxSkp2. Rbx1 binds the E2–Ub conjugate. The target protein binds to the F-box protein that is bound to the Skp1 subunit that, in turn, is connected to the Cul1–Rbx1 enzyme core [99]. It has been demonstrated that the levels of Cullin1 (Cul1) and Skp1, both of which are core components of the SCF complex, are reduced in HD mouse brain. In addition, Cul1 silencing results in an increased aggregate load and enhanced polyQ-induced toxicity in cultured cells and in Drosophila. These results imply that reduced levels of the SCF complex might contribute to polyQ disease pathology [85].

## 6. E3 Ligases Counteracting the Effect of mHtt on Oxidative Metabolism

### HACE1 Reduces Oxidative Stress and mHtt Toxicity

HECT domain- and ankyrin repeat-containing E3 Ub protein ligase 1 (HACE1), known to play a protective role against stress-induced tumorigenesis in mice, also play a role in antioxidative stress response and, as a consequence, in neurodegeneration.

HACE1 KO mice exhibit increased oxidative stress in the brain and a reduced expression of NQO1, a marker of antioxidative stress response, and of the NRF2 (nuclear factor-erythroid 2-related factor 2) transcription factor. Thus, the consequences of HACE1 deficiency are similar to those measured in the absence of NRF2, indicating that HACE1 positively regulates the NRF2-mediated antioxidative stress response and has a fundamental role in maintaining redox homeostasis in brain tissue. In particular, HACE1 levels are decreased in mHtt-expressing STHdhQ111 cells, which correlates with the diminished activity of NRF2 and the increased oxidative stress. On the other hand, ectopic HACE1 expression in STHdhQ111 cells increases NRF2 activity, thereby providing protection against mHtt-induced redox imbalance and hypersensitivity to oxidative stress. Increasing HACE1 expression may therefore represent a strategy to counteract the consequences of HD and, possibly, other neurodegenerative diseases on redox metabolism [86].

## 7. E3 Ub Ligases Increasing mHtt Levels

### 7.1. WWP1 Inhibits mHtt Degradation

NEDD4-like E3 Ub protein ligase (also known as WWP1) is a 922-residue HECT E3 ligase that promotes the ubiquitination and internalization of various plasma membrane channels, such as ENaC, SCN2A/Nav1.2, SCN3A/Nav1.3, SCN5A/Nav1.5, SCN9A/Nav1.7, SCN10A/Nav1.8, KCNA3/Kv1.3, KCNH2, EAAT1, KCNQ2/Kv7.2, KCNQ3/Kv7.3, or CLC5 [100,101]. The X-ray crystal structures of several protein domains have been solved to date (see Table 2). WWP1 has been shown to be involved in mHtt ubiquitination. mHtt upregulates WWP1, which is overexpressed in R6/2 mice and in N2a cells expressing mHtt. In contrast to UBE3A and the SCF complex, WWP1 was shown to increase mHtt levels in the cell and, as a consequence, contribute to neuron deterioration and death. This occurs because WWP1 catalyzes the ubiquitination of mHtt via K63 and not via K48, thereby promoting mHtt oligomerization and inhibiting mHtt degradation via the proteasome [87].

### 7.2. TRAF6 Ubiquitinates mHtt Fragments Inducing Aggregate Formation

Tumor Necrosis Factor Receptor-Associated Factor 6 (TRAF6) is a 522 residue protein structurally belonging to the RING-type E3 ligase family. It has an important role in the receptor-mediated activation of various signaling pathways in response to cytokines and bacterial products. TRAF6 also possesses E3 Ub ligase activity that results in Lys-63-linked polyubiquitination of target proteins [102,103].

Among these, TRAF6 mediates the polyubiquitination of proteins involved in immune signal activation via K63, such as inhibitor of nuclear factor kappa-B kinase subunit gamma (IKBKG), interleukin-1 receptor-associated kinase 1 (IRAK1), AKT1, and AKT2, thereby modulating the immune and inflammation responses [104]. TRAF6 was also found to interact, without preference, with N-terminal fragments of mutated and wild-type Htt and catalyze their ubiquitination via K6, K27, and K29 [88]. In other cases, such unconventional ubiquitination was found to regulate cell signaling, intracellular trafficking, and biochemical activities [105]. TRAF6-dependent ubiquitination of mutated Htt fragments induces the formation of aggregates, which have been found in the brain of HD mouse models [88].

## 8. PROTACs

**Figure 5:**
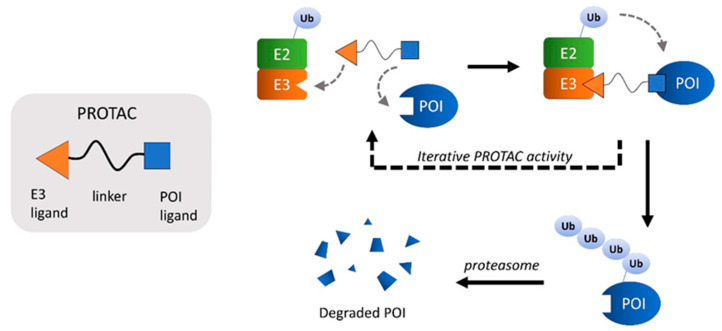
General mechanism of action of PROTACs. PROTACs are drug-like compounds comprising two active ends joined by a linker. The ends are specific ligands able to recognize and “hook” an E3 ligase and a protein of interest (POI). Upon binding, PROTAC induces the formation of the ternary complex E3 ligase/E2-Ub/POI, thus favoring POI ubiquitination and, consequently, degradation by the proteasome.

Proteolysis targeting chimeras (PROTACs) are heterobifunctional small molecules composed of two active moieties joined to each other via an appropriate linker; one of the moieties binds a target protein of interest (POI), the other binds an E3 Ub ligase (Figure 5). PROTACs induce proximity between the POI and the E3 ligase, which, in turn, interacts with E2–Ub. This leads to the formation of a ternary complex that allows POI ubiquitination and subsequent degradation by the proteasome. Most of the PROTACs synthesized during the past few years are able to recruit one of the following three proteins, all of which are part of E3 ligase complexes: von Hippel–Lindau protein (pVHL), cereblon (CRBN), and inhibitors of apoptosis proteins (IAPs) [106,107,108,109].

pVHL is a subunit of the E3 Ub ligase CUL2–RBX1–ElonginB–ElonginC–VHL (CRL2VHL) complex. pVHL is responsible for the recognition and binding of hypoxia-inducible factor-1α (HIF1-α factor), which controls a series of biological processes, such as ROS production, angiogenesis, and cancer metastasis. Upon pVHL binding, the HIF1-α factor is ubiquitinated for proteasomal degradation. The crystal structure of the pVHL–ElonginB–ElonginC complex, bound to the C-terminal oxygen-dependent degradation (CODD) motif of HIF1α, has allowed the pVHL structural features required to bind a specific hydroxyl proline residue present on CODD to be identified [104,110,111]. As a consequence, the first PROTAC synthesized to bind pVHL consisted of a peptide able to bind the CODD motif (ALAPYIP) and conjugated: at the N-terminus to a ligand able to bind the target protein via a linker; at the C-terminus to a poly-D-arginine tag to confer cell permeability [112]. Since peptides are prone to be hydrolyzed very easily, this first PROTAC was substituted by peptidomimetic molecules. Second-generation ligands were later developed via structure-based drug design, and using co-crystal structures and isothermal titration calorimetry (ITC) to optimize the interactions of the VHL ligand with a rational, stepwise approach [113]. This optimization strategy led to the development of strong VHL binders with nanomolar binding affinity. One of these VHL ligands, VH032, has been used to design the first VHL-based PROTAC, i.e., MZ1, which causes the degradation of bromodomain protein BRD4 in cancer cells [107].

**Figure 6:**
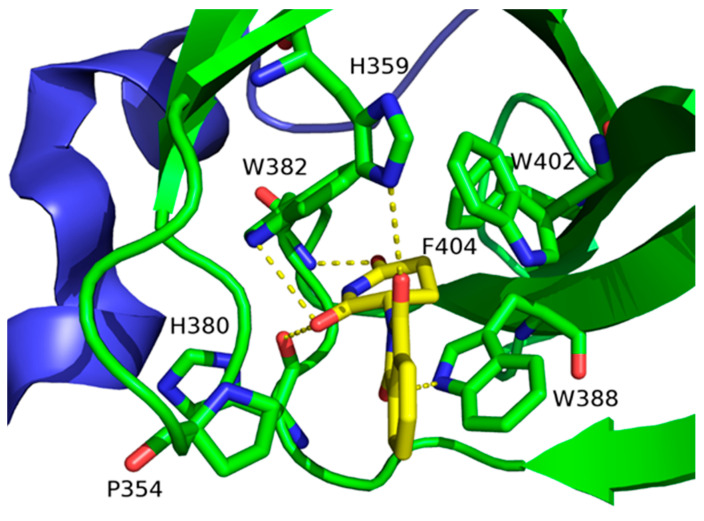
X-ray structure of the complex between thalidomide and CRBN (PDB code: 4CI1). Thalidomide and CRBN residues interacting are represented as sticks and colored by atom type (N, blue; O, red; C, yellow and green for thalidomide and CRBN, respectively). Other residues are displayed as ribbon and are colored green in the thalidomide binding domain, and blue in the rest of CRBN.

Cereblon (CRBN) forms an E3 Ub ligase complex with damaged DNA binding protein 1 (DDB1), Cullin-4A (CUL4A), and Regulator of Cullins 1 (ROC1) [114]. CRBN is the major target of thalidomide. Phthalimides binding to CRBN leads to the formation of an interface that facilitates the recruitment of Ikaros family transcription factors zinc finger protein (IKZF)-1 and -3 for proteosome degradation. The X-ray structures of DDB1–CRBN–thalidomide complexes revealed that a portion of the phthalimide ring of thalidomide is exposed to solvent and can be exploited to add a substituent or linker (Figure 6) [115,116]. On this basis, PROTACs dBET1, ARV825, and dFKBP were developed, which exert tumor growth inhibition activity by promoting the degradation of BET proteins or FKBP12 [106,117]. The Arvinas company has recently presented provisional results of a Phase 1/2 clinical trial of their ARV-110 PROTAC, a Selective Androgen Receptor Degrader (SARD) whose structure is still undisclosed, which targets the androgen receptor in men with metastatic castration-resistant prostate cancer (mCRPC) [118]. ARV-471 is a PROTAC similar to ARV-110, which targets the estrogen receptor [119]. Both ARV-110 and ARV-471 bind to CRBN and feature a short, rigid, nitrogen-containing linker, which is markedly different from the flexible linkers used in early, academic versions of protein degraders.

IAP family members are characterized by the presence of three baculovirus IAP repeat (BIR) domains at the N-termini, and they act as apoptosis inhibitors. Some of these proteins, such as cellular inhibitor of apoptosis-1 (cIAP1) and X-linked inhibitor of apoptosis protein (XIAP), contain a RING domain and are able to function as E3 ligases. It was reported that methyl bestatin binds to the BIR3 domain of cIAP1, thereby inducing self-ubiquitination and degradation. PROTACs made of bestatin, all-trans retinoic acid (ATRA), and a polyethylene glycol (PEG) linker were found to successfully induce the degradation of cellular retinoic acid binding protein-2 (CRABP2) and have been called SNIPERs (Specific and Nongenetic Inhibitor of Apoptosis Protein (IAP)-Dependent Protein Erasers) [109,120]. One characteristic feature of SNIPERs is that they can retain self-ubiquitination activity toward cIAP1, a synergistic activity for the therapy of those types of cancer cells in which cIAP1-induced degradation is beneficial.

Other recent works have expanded the toolbox for PROTACs, such as the Kelch-like ECH-associated protein-1 (KEAP1), which is able to form a complex with Cullin-3 (CUL3) E3 ligase, to bind and induce the ubiquitination and degradation of Nrf2 [121]. Both peptidic and non-peptidic PROTACs recruiting the KEAP1–CUL3 E3 ligase have been synthesized and were shown to degrade Tau [122] and BRD4 [123], respectively.

Another protein which acts as an atypical substrate-specific component of CUL4B-based E3 complex is the arylhydrocarbon receptor (AhR) transcription factor. Ohhoka and coworkers synthesized PROTACs able to bind AhR, thereby inducing the AhR-dependent degradation of CRABP-1 and CRABP-2 via the ubiquitin–proteasome pathway [124]. Other groups were able to synthesize PROTACs against the RING-type E3 ligases RNF4 [125] and RNF114 [126].

### 8.1. Targeting the Ubiquitination Pathways for the mHtt Clearance

The discovery of novel PROTAC protein degraders is one of the most promising approaches for neurologic diseases, which have been historically difficult to treat. This is partly due to the difficulty of crossing the blood–brain barrier and delivering therapeutic molecules to the brain, and to the fact that many of the targets within the central nervous system (CNS) are considered to be ‘undruggable’. Arvinas and other companies are developing PROTAC protein degraders as potential therapeutics for neurodegenerative diseases.

PROTACs have important advantages with respect to other therapies, including: the ability to cross the blood–brain barrier (BBB), which is a substantial challenge for monoclonal antibodies and genomic therapies; the ability to enter cells of the CNS, which may allow pathogenic proteins to be degraded inside the cell; iterative (often described as ‘catalytic’) activity by which a single PROTAC molecule can induce the degradation of hundreds of copies of disease-causing proteins; potential for oral dosing unlike other therapies that may require intrathecal or intravenous (IV) administration.

One of the ideas that has been explored for HD therapy consists of the elimination of mHtt through the UPS by increasing mHtt polyubiquitination via K48 [127]. For this purpose, a PROTAC was synthesized using the bestatin derivative BE04, which is a specific ligand of cellular inhibitor of apoptosis protein 1 (cIAP1), and conjugated with benzothiazole derivative (BTA) or phenyldiazenyl benzothiazole derivative (PBD), which are probes for protein aggregates, and aimed at directly targeting mHtt aggregates [127]. Another PROTAC was synthesized based on MV1, an IAP antagonist, which is likely to have a higher affinity for cIAP1 than BE04 [108]. In both cases, both Htt and mHtt levels were decreased in fibroblasts from both HD patients and healthy individuals. These two PROTACs were demonstrated to be able to degrade not only mHtt but also other β-sheet structures in mutated polyQ-containing proteins responsible for diseases, such as ataxin-3 and ataxin-7 in fibroblasts from spino-cerebellar ataxia (SCA) patients, and atrophin-1 in fibroblasts from entatorubral–pallidoluysian atrophy (DRPLA) patients [128].

### 8.2. Targeting the Autophagosomal Pathway to Reduce mHtt

The use of molecules that interact with both mHtt and the proteins of the autophagy-lysosomal pathway (which is outside of the scope of the present review but is worth mentioning in this context) is another possible strategy for degrading mHtt. Compounds interacting with both polyQ stretches and the autophagosome protein microtubule-associated protein 1A/1B light chain 3 (LC3) have been discovered by high-throughput screening (HTS). Four of these compounds, i.e., AN1, AN2, 10O5, and 8F20, were found to be able to lower the level of both mHtt and ataxin-3 by targeting them to the autophagosomes [129].

## 9. Conclusions

The polyubiquitination of proteins is a pathway that controls protein expression and degradation. Polyubiquitination involves the side chains of the seven Ub lysine residues and the free main-chain of the N-terminal methionine residue and can be either homotypic or heterotypic. The specific type of polyubiquitination determines the fate of the target protein.

Monoubiquitinated mHtt can be polyubiquitinated via either K48 or one of the other Ub lysine residues (K6, K11, K27, K29, K33, K48, and K63). The polyubiquitination of mHtt and its fragments via K48 ensures their clearance through the proteasome complex, whereas polyubiquitination via K63 promotes aggregate formation.

Several E3 ligases that are involved in the polyubiquitination of Htt and Htt fragments via K48, promoting their degradation via proteosome, have been identified and described in this review (i.e., UBE3A, CHIP, Hrd1, Parkin, and SCF complex). In particular, UBE3A expression decreases with age in both healthy mice and the R6/2 mouse model, and the diminished expression of UBE3A in neurons is strongly related to aging and to the worsening of the HD phenotype.

**Figure 7:**
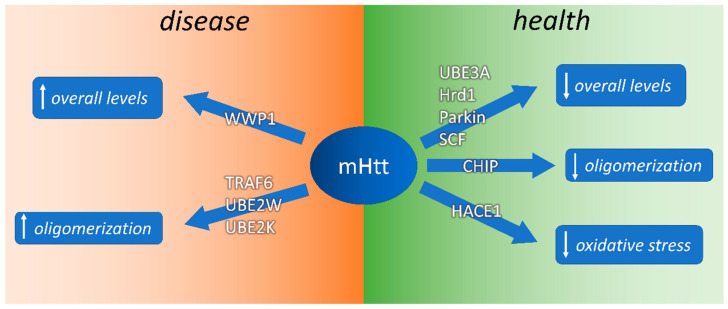
Ubiquitin–proteasome system enzymes involved in mHtt ubiquitination and polyubiquitination and their effects on HD pathology.

Conversely, WWP1 and TRAF6 E3 ligases, which polyubiquitinate mHtt via K63 of mHtt-linked ubiquitin, are overexpressed in R6/2 mice. As a consequence, the UPS system becomes highly unbalanced upon aging, with uncontrolled increase in polyubiquitinated mHtt, which remains free in the cells (Figure 7).

All current strategies to cure HD by decreasing mHtt concentration are based on antisense oligonucleotides (ASOs), which target mRNA of both mHtt and Htt, or mHtt alone, in cells. Different ASO-based drugs have entered clinical trials, and some of them have already failed in phase III (i.e., tominersen by Roche) or in phase II clinical trials (i.e., WVE-120101 and WVE-120102 by Wave Life Sciences). The reasons underlying these failures have not yet been disclosed. However, all these therapies require drug delivery by lumbar punctures to be administered at least every two months.

Due to their effectiveness and the relative facility and velocity of implementation, strategies aimed at modulating mHtt metabolism by selectively enhancing mHtt ubiquitination by PROTACs appear to be advantageous for HD therapy. Among the targets that can be exploited to design lead compounds aimed at decreasing soluble mHtt concentration, UBE3A appears to be particularly promising because it is the E3 ligase that normally polyubiquitinates Htt via K48 and has a key role in the control of mHtt concentration in the cell. The development of PROTACs targeting both UBE3A and mHtt is particularly attractive due to the possibility to rationally design effective ligands of both proteins based on the three-dimensional structures of UBE3A catalytic domain and wild-type Htt available from the Protein Data Bank (www.rcsb.org).

## Figures and Tables

**Table 1:** List of ubiquitinated and SUMOylated residues in human Htt, according to PhosphoSitePlus (phosphosite.org).

Residue (Human Htt)	Demonstrated Modification	Reference
Lys 6	SUMOylation	[66]
Lys 9	SUMOylation	[66]
Lys 253	Ubiquitination	[67]
Lys 335	Ubiquitination	[68]
Lys 442	Ubiquitination	[69]
Lys 662	Ubiquitination	[68]
Lys 667	Ubiquitination	[67]
Lys 698	Ubiquitination	[67]
Lys 813	Ubiquitination	[67]
Lys 902	Ubiquitination	[67]
Lys 937	Ubiquitination	[67]
Lys 941	Ubiquitination	[70]
Lys 1121	Ubiquitination	[68]
Lys 1223	Ubiquitination	[68]
Lys 1244	Ubiquitination	[68]
Lys 1262	Ubiquitination	[71]
Lys 1337	Ubiquitination	[67]
Lys 1402	Ubiquitination	[67,68,70,71,72]
Lys 1410	Ubiquitination	[67,68,70]
Lys 1415	Ubiquitination	[68]
Lys 1431	Ubiquitination	[68,70,73]
Lys 1885	Ubiquitination	[67]
Lys 2417	Ubiquitination	[71]
Lys 2423	Ubiquitination	[68]
Lys 2443	Ubiquitination	[68]
Lys 2537	Ubiquitination	[67]
Lys 2564	Ubiquitination	[67]
Lys 2757	Ubiquitination	[67]
Lys 2901	Ubiquitination	[67]
Lys 2967	Ubiquitination	[67]

**Table 2:** Ubiquitin–proteasome system enzymes involved in mHtt and Htt ubiquitination and polyubiquitination.

Protein	Function/Type	Effect on mHtt	Protein Length	PDB Codes
UBE2K/E2-25k	E2	mHtt oligomerization	200 residues	1YLA (1–200), 2O25 (1–200), 3E46 (1–200), 3F92 (1–200), 3K9O (1–200), 3K9P (1–200), 5DFL (1–200), 6IF1 (1–199), 6JB6 (1–200), 6JB7 (1–200)
UBE2W	E2	mHtt oligomerization	151 residues	2A7L (1–117), 2MT6 (1–151)
UBE3A/E6AP	HECT-type E3	mHtt clearance	875 residues	1C4Z (518–875), 1D5F (518–875), 1EQX (401–418), 2KR1 (24–87), 4GIZ (403–414), 4XR8 (406–417), 6SJV (403–417), 6SLM (403–417), 6TGK (765–869), 6U19 (24–87)
CHIP/STUB1	U-box E3	mHtt oligomerization inhibition	303 residues	4KBQ (21–154), 6EFK (23–154), 6NSV (23–152)
HRD1/SYVN1	RING-type E3	httN clearance	617 residues	6A3Z (279–334), 6JB7 (1–200)
Parkin	RING/HECT hybrid E3	mHtt clearance	465 residues	1IYF (1–76), 2JMO (308–384), 4BM9 (137–465), 4I1F (141–465), 5C1Z (1–465), 5C23 (1–465), 5C9V (137–465), 5N2W (1–465), 5N38 (1–465), 5TR5 (1–76), 6GLC (1–382), 6HUE (1–465), N13 (144–465)
SCF complex (formed by Rbx1, Cul1, Skp1)	E3 complex	mHtt clearance		1LDJ
HACE1	HECT-type E3	NRF2-mediated antioxidative stress	909 residues	No structure
WWP1	NEDD4-like E3	Increase in mHtt in the cell	922 residues	1ND7 (546–917), 2OP7 (494–532), HPS (537–917), 5HPT (537–917), 6J1X (379–922), 6J1Y (410–922)
TRAF6	RING-type E3	Increase in mHtt fragment aggregates	522 residues	1LB4 (348–504), 1LB5 (347–504), 1LB6 (347–504), 2ECI (50–128), 2JMD (67–124), 3HCS (50–211), 3HCT (50–159), 3HCU (50–159), 4Z8M (346–504), 5ZUJ (350–501), 6A33 (350–501), 7L3L (52–158)

Numbers in parentheses after PDB codes indicate the residues present in each PDB file. mHtt: mutant huntingtin.
